# Predicting cognitive performance from physical activity and fitness in adolescents and young adults in Botswana relative to HIV status

**DOI:** 10.1038/s41598-019-55925-x

**Published:** 2019-12-20

**Authors:** Leapetswe Malete, Jennifer L. Etnier, Dawn M. Tladi, Jarod C. Vance, Gabriel M. Anabwani

**Affiliations:** 1 Department of Kinesiology, Michigan State University, East Lansing, Michigan USA; 2 Department of Kinesiology, University of North Carolina at Greensboro, Greensboro, North Carolina USA; 3 Department of Physical Education, Health & Recreation, University of Botswana, Gaborone, Botswana; 4 The Botswana-Baylor Children’s Clinical Centre of Excellence, Gaborone, Botswana

**Keywords:** Psychology, Diseases

## Abstract

Little is known about whether physical activity and fitness could enhance cognition in adolescents and young adults living with HIV. The purpose of this study was to examine this relationship in a group of 250 HIV+ (n = 88) and HIV negative (n = 162) participants from Botswana, aged 12–23 years (Mean = 17.87, SD = 2.24). Fitness was operationalized as muscular strength (push-ups) and aerobic endurance (PACER). PA was assessed using items from the Youth Risk Behavior Surveillance Survey. Cognition was measured using the Corsi Test, Berg Card Sorting Task (BCST), and Stroop Color Word Task (Stroop). Multiple regression analyses indicated that the HIV x push-ups interaction was a significant predictor of Corsi performance, and HIV status was a significant predictor of BCST performance (p < 0.05). For the Stroop portions, HIV status and HIV x push-ups were significant predictors (p < 0.01). HIV status is predictive of cognition and interacts with muscular fitness to predict cognition.

## Introduction

An estimated 36.9 million people were living with Human Immunodeficiency Virus (HIV) throughout the world and 1.8 million people became newly infected in 2017^1^. Sub-Saharan Africa accounted for 71% of all people living with HIV (HIV+) even though only 12% of the global population live in this region^2^. In Botswana, the prevalence of HIV for 15–24 year olds was 6% for females and 3.5% for males and in other Sub-Saharan African countries prevalence was as high as 13.1%^2^. The increase in availability of antiretroviral therapy (ART) has resulted in a significant decline in mortality among HIV+ individuals^3^. Despite this success, HIV-associated neurocognitive disorders (HAND) are prevalent^4–6^. Therefore, a growing body of research has begun to examine how physical activity (PA) and exercise may be capable of reducing HAND symptoms^7–10^. To date, the majority of this research has focused on middle-aged and older adults, has been conducted on populations in the United States^7,8,10^, or has had very large age ranges (e.g. 18–60 years) and small samples^9^. While there has been an increase in the number of studies on HAND and mental health outcomes among youth living with HIV in African contexts, an overwhelming number involve Ugandan children and youth populations^5,6,11,12^. The studies offer an excellent foundation for research into cognitive function for children and adolescents living with HIV, but none are known to address the relationship between PA, fitness, and cognitive function specifically in adolescents.

The potential benefits of PA and fitness have been explored for children and adolescents who are physically healthy. In an early meta-analytic review, Sibley and Etnier^13^ reported that PA that ranges from acute (single session) to chronic (multiple sessions over a period of weeks or months) has an overall significant but small benefit (Hedge’s *g* = 0.32) for cognitive performance. More recently, Donnelly *et al*.^14^ conducted a systematic review of the literature for the Position Stand of the American College of Sports Medicine. Accordingly, cross-sectional studies focused on fitness and generally supported a positive relationship with cognitive performance. Further, randomized controlled trials were also found to consistently report benefits to cognitive performance for the exercise group that exceeded any changes observed for the control group. While there is evidence that support the benefits of regular PA to cognitive performance among children and adolescents in general, no studies to our knowledge, have been conducted to examine the relationships between PA, fitness, and cognition among adolescents and young adults relative to HIV status.

Hence, it is critical to understand the potential negative impact of HIV on the cognitive performance of these individuals as well as the role of PA in attenuating the impact. If adolescent and young adult living with HIV face an increased risk for early decrement in cognitive performance, early interventions may attenuate HIV-associated decline in cognitive functioning. Therefore, the main purpose of this study was to examine the relationship between self-reported PA behaviors, fitness level, and cognitive functioning in an HIV+ population of young adults and adolescents in Botswana. We hypothesized that HIV+ participants would perform significantly worse on cognitive tasks compared to healthy controls. Further, HIV+ participants who take part in more PA behaviors and have greater levels of fitness would also exhibit greater cognitive functioning in comparison to HIV+ participants who are less active or have lower levels of fitness.

## Methods

### Participants

A total of 250 HIV+ and HIV− adolescents and young adults (138 females) aged 12–23 years (Mean = 17.87, SD = 2.24) participated in the study. The HIV+ group (n = 88) was part of a previous 12-month ART and nutrition randomized controlled trial involving 201 participants. Participants in this group were recruited into the first study at the time of enrollment in an ART program offered by a pediatric clinic in Gaborone, Botswana. Eligibility to enroll in that study was based on HIV positive results from an ELISA test; living within 100km of Gaborone; taking first- or second-line ART regimen; having a low CD4 count (between 100–200 cells/mm^3^); viral load at or below 500 copies/mL; and being on first- or second-line ART. Patients with an active AIDS-defining illness or an active but untreated HIV/AIDS-related illness were excluded from the study. All participants were infected with subtype C virus prenatally or soon after birth via mother-to-child transmission. Their pre-morbid cognitive conditions were not known.

The treatment was an ART and nutrition intervention. Based on records of monthly visits to the pediatric clinic and the study center over the 12 months of the study, the HIV+ participants had very high adherence to the treatment regimen and the study’s protocol. They experienced a significant increase in their CD4 cell count and a decrease in viral load over the 12-month period. This data and other clinical indicators are provided in Table 1. The trends are in line with findings of a national study that showed high adherence rates, near normal to normal immunological states in 86.6% of program participants, and undetectable virus (<400 copies/m) in 92% of them^15^.

**Table 1:** Selected clinical data (Mean ± SD) for the HIV+ group at baseline and after the 12 months ART and nutritional intervention study.

Variable	Baseline	12 months	p-value^a^
CD4 cell count/mm^3^	591.4 ± 283.9	703.8 ± 394.5	p < 0.001
CD4%	23.5 ± 7.6	27.0 ± 7.7	p < 0.001
Viral load (log)	14031.7 ± 67795.7	3110.8 ± 10514.2	p < 0.001
Hemoglobin (g/dL)	11.8 ± 1.2	12.1 ± 1.1	p = 0.01
Prealbumin (g/L)	0.17 ± 0.1	0.22 ± 0.1	p = 0.21
C-reactive protein (mg/L)	11.3 ± 21.2	9.9 ± 15.8	p = 0.85
Presents with ongoing symptoms	45 (51%)	7 (7%)	

^a^Paired t-test (Baseline vs. 12 months).

Participants who could be contacted were invited to participate in the present study. Out of the possible 200 HIV+ participants, 88 agreed to take part. Twenty could not take part because of various constraints including living too far away from the study location and others could not be found through their contact information. The HIV− group (n = 162) was a randomly selected cohort of public secondary (junior and senior high) school students from school districts in Gaborone. There was not a significant difference in the proportion of female to male participants in the HIV+ group (44:43) as compared to the HIV− group (94:68), X^2^(1) = 1.27, p > 0.05; however, there was a significant difference in age between the groups, *t*(225.803) = 4.56, *p* < 0.001. The HIV− status of this cohort was verified by a national HIV testing and counselling center prior to enrollment in the study. A summary of the sample’s key demographic variables is provided in Table 2.

**Table 2:** Subjects’ demographic and health information.

Variable	Total N = 250	HIV+ N = 88	HIV− N = 162
Age	17.87 ± 2.24	18.65 ± 1.74	17.45 ± 2.36^
Height (cm)	161.69 ± 9.69	159.79 ± 9.18	162.71 ± 9.82^
Body Mass (kg)	52.15 ± 9.49	49.26 ± 6.97	53.70 ± 10.29^
BMI (kg/m^2^)	19.99 ± 3.78	19.37 ± 3.08	20.33 ± 4.07
Diabetes, n (%)	29 (12%)	18 (21%)	11 (7%)*
Elevated Cholesterol, n (%)	1 (0.4%)	0	1(0.6%)
Hypertension, n (%)	1 (0.4%)	0	1 (0.6%)
Asthma, n (%)	16 (6%)	10 (11%)	6 (4%)*
Chest pains, n (%)	6 (2%)	4 (5%)	2 (1%)
Bone injury, n (%)	24 (10%)	8 (9%)	16 (10%)
Allergies, n (%)	18 (7%)	9 (10%)	9 (6%)
Epilepsy, n (%)	29 (12%)	15 (17%)	14 (9%)

*p-value < 0.05 Pearson Chi square test.

^^^p-value < 0.05 Independent sample t-test.

### Measures

#### Demographics questionnaire

A questionnaire was used to gather demographic information such as age, sex, and school level. Socioeconomic status (SES) was determined through responses to a questionnaire item on indoor plumbing. Participants were coded as having higher SES if they reported having indoor plumbing and lower SES if they did not report having indoor plumbing. The rationale for using indoor plumbing as a proxy for SES was that, compared to other commonly used indicators such as income, assets, and education, indoor plumbing is a more objective common denominator of low-income status in developing countries. It may also serve as a good indicator of income, assets, and level of education, while assets and income tend to be unreliable due to recall.

#### Daily aerobic PA and muscle strengthening

Daily aerobic PA and muscle strengthening were assessed using self-report, paper pencil items from the Centers for Disease Control and Prevention’s (CDC) Youth Risk Behavior Surveillance Survey (YRBSS)^16^. First participants were asked to indicate how many days out of the past seven they were active for a total of 60 minutes in moderate-to-vigorous aerobic PA (MVPA). The same was done for exercises aimed at strengthening or toning muscles, such as push-ups, sit-ups, or weightlifting. This variable was labelled muscular strengthening activity (MSA). The MVPA and MSA items have been widely used in population-based surveys of children, adolescents, and young adults^17–19^.

#### Fitness

Fitness was operationalized as muscular strength (number of push-ups completed) and aerobic endurance (PACER). The PACER test has been used in numerous settings to assess children and adolescent’s aerobic fitness level and has been found to be valid^20–22^, have high test-retest reliability^22,23^, and be an accurate measure of aerobic capacity^24,25^.

#### Body mass index (BMI)

Weight and height were measured using a portable digital scale (Tanita, Tokyo, Japan) accurate to 0.1 kg and a stadiometer (Seca, Hamburg, Germany) accurate to 1 mm and BMI (kg/m^2^) was calculated.

### Measures of cognition

The Psychology Experiment Building Language program was used for cognitive task implementation. The Corsi Test (working memory), Berg Card Sorting Task (BCST; planning), and Stroop Color Word Task (processing speed and inhibition) were the measures of cognition, and these have been validated in adolescents through adults^26–28^. Cognitive measures focused on executive function because of evidence that these aspects of cognition are sensitive to the effects of PA in children^29^.

#### Stroop test

**Figure 1:**
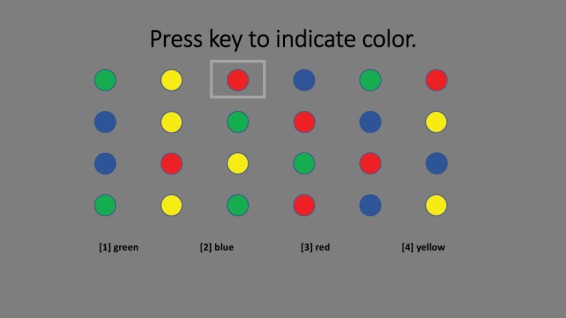
Stroop Color Task.

**Figure 2:**
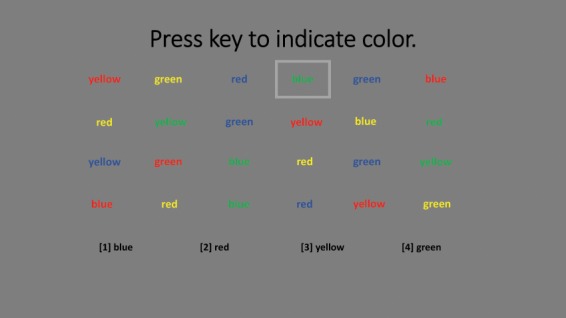
Stroop Color-Word Task.

In this version of the Stroop test, participants see 24 stimuli (6 across x 4 down) displayed at the top of the screen. They are instructed to use the numbers on the keypad of 1, 2, 3, and 4 to indicate the color of the stimulus as red, green, blue, or yellow, respectively. The legend showing the link between the number and the color is always available at the bottom of the screen. A square outlines the current stimulus. A correct answer results in the square advancing to the next stimulus. An incorrect answer results in the square refreshing. The Stroop Color Task and Stroop Color-Word tasks are illustrated in Figs. 1 and 2.

Participants completed 24 trials of each task. For the Stroop Color Task, the stimuli were circles colored red, green, blue or yellow. For the Stroop Word Task, the stimuli were non-color words (e.g., over, when) written in colored ink. For the Stroop Color-Word Task, the stimuli were color names (red, green, blue, yellow), written in ink of a different color. Performance was recorded as total response time included time lost due to erroneous answers. The Stroop Color and Word tasks are considered information processing tasks while the Color-Word task requires inhibition and is considered a measure of executive functioning. Total response time to complete the 24 trials was measured for each of the three blocks.

#### Corsi Test

**Figure 3:**
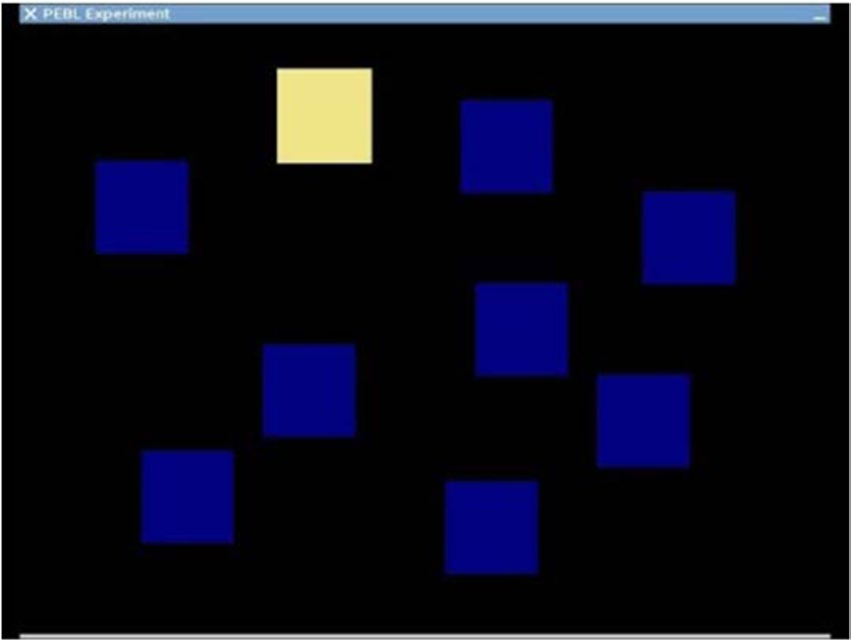
Corsi Test.

Participants see a display of nine blue blocks on the screen. The blocks light up (turn from blue to yellow) in a particular order, then the participant is asked to use the mouse to click on the blocks in the same order. Feedback is provided as to whether they are correct or incorrect. They do 3 practice trials of three blocks each. They then start with 2 blocks to remember and have 2 trials at that level. If they get at least one right, the sequence that must be remembered increases by one. If they miss both trials at the same sequence length, the program stops. Performance is assessed as the longest number of blocks they could remember in order on at least one trial. The Corsi Test provides a measure of spatial working memory. Block Span was computed as the longest length at which at least one pattern was correctly recalled consecutively. Total correct trials were the number of total trials that were correctly recalled. The Corsi Test is illustrated in Fig. 3.

#### Berg Card Sorting Task-64

**Figure 4:**
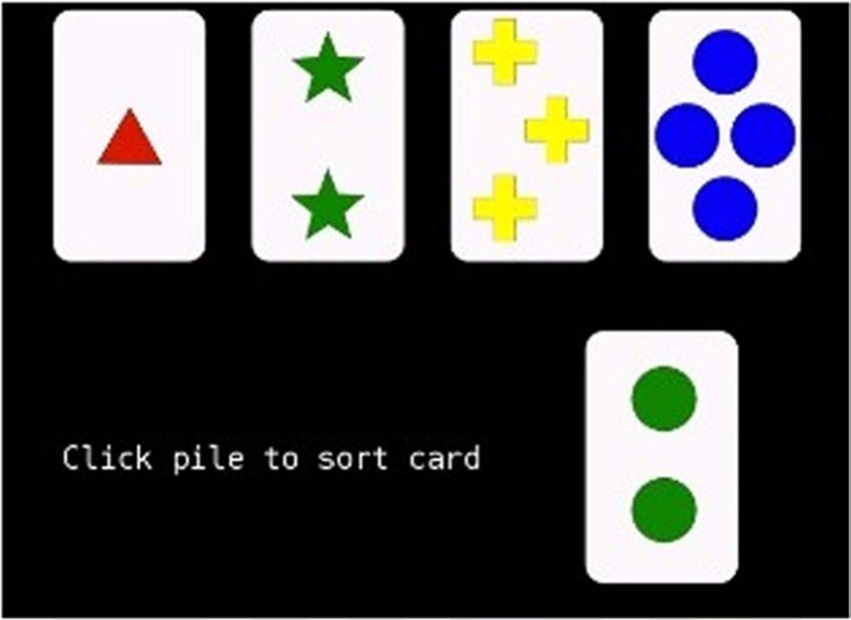
The Berg Card Sorting Task-64.

For this task, participants see 4 “cards” at the top of the screen. The cards are a single red triangle, two green stars, three yellow plus signs, and four blue circles. Participants are instructed to use the mouse to identify which card is a match for the stimulus card that they are shown. The rule can be to match based upon color, shape, or number. They must figure out the “rule” governing a match by paying attention to the feedback of “correct” or “incorrect” that is provided. The 64 stimulus cards have 1–4 shapes (triangle, star, plus sign, circle) that are in one of the four colors. Scores used from this task included the number of correct responses, the number of trials to complete the first set of 10 correct in a row, the number of perseverative responses, and the number of perseverative errors. This task provides a measure of executive functioning. The total correct was calculated by summing the total correct responses (correct = 1, incorrect = 0). Perseverative errors were calculated by summing the perseverative errors made (responses made following the old rule). Trials to complete first set was the total number of trials completed before the run switched to the second set. The Berg Card Sorting Task-64 is illustrated in Fig. 4.

### Ethical clearance

Permission to conduct this research was obtained from the Ethics Committee of the Ministry of Health in Botswana. Additional IRB approvals were obtained from the collaborating institutions in Botswana. All methods were performed in accordance with the relevant guidelines and regulations. Participants who were willing to participate in the study were given an introductory letter, informed consent forms, and assent forms to take home for their parents or guardians to read and sign. They were advised to return the signed forms on the day of data collection. The letter and consent forms specifically made participants and their parents and /or legal guardians aware of how the information provided would be used and protected, as well as participants rights to decline participation, withdraw from the study at any point, or decline to answer any questions. All participants provided signed informed consent forms prior to taking part in the study’s activities. Participants under the age of 18 years provided signed informed consent forms from their parents or legal guardians as well as their own assent forms before participating in the study.

### Data reduction

Prior to running analyses for the Stroop data, analyses of variance (ANOVAs) were conducted for each condition of the Stroop test to examine differences in total response time for correct trials between the two HIV status groups. Results showed that the two groups were statistically different on total response time for the color condition, F(1,5038) = 15.08, p < 0.001, and for the word, F(1,5014) = 168.87, p < 0.001, and color/word conditions, F(1,5014) = 201.11, p < 0.001. Since the groups were different in performance, outliers (2 standard deviations from the mean) were identified separately for each group and excluded from subsequent analyses. For both HIV status groups and all Stroop conditions, this resulted in the exclusion of between 0.13–2.81% of trials.

### Statistical analyses

Preliminary analyses were performed to ensure there was no violation of the assumption of normality, linearity, multicollinearity, and homoscedasticity. To examine the predictive relationship of sex, age, SES, HIV status, PA behaviors, and fitness on cognitive performance (Corsi, BCST, Stroop), hierarchical multiple regressions were conducted. Continuous predictor variables (PACER, push-ups, MVPA, and MSA) were centered at the mean. Model 1 included age, sex and SES to control for these potential confounds. Models 2 and 3 tested for main effects. Model 2 included HIV status, and Model 3 included PACER, push-ups, MVPA, and MSA. Model 4 tested for interactions of HIV status × MVPA, HIV status × MSA, HIV status × PACER, and HIV status × push-ups. The data was analyzed using SPSS (IBM SPSS Statistics Version 25).

## Results

### Corsi Test

For model 1, 7.8% of the variance was explained for Corsi total correct trials, F(3, 212) = 5.97, p < 0.01. After entry of HIV status, model 2 was significant, F_change_(1, 211) = 8.52, R^2^_change_ = 4%, p < 0.01. For model 3, PACER, push-ups, MVPA, and MSA were added and found to be significant, F_change_(4, 207) = 2.67, R^2^_change_ = 4%, R^2^ = 15.7%, p < 0.05. Model 4 was non-significant. In the final significant model, sex (β = 1.13, CI_95%_: 0.26–2.00, p = 0.012), HIV status (β = −0.74, CI_95%_: −1.43 to −0.05, p = 0.036), and push-ups (β = 0.043, CI_95%_: 0.01–0.07, p = 0.008) were significant predictors.

For model 1, 9.7% of the variance was explained for Corsi block span, F(3, 211) = 7.52, p < 0.001. After entry of HIV status, model 2 was significant, F_change_(1, 210) = 6.46, R^2^_change_ = 3%, p < 0.05. Model 3 was not significant. Model 4 was significant, F_change_(4, 202) = 2.50, R^2^_change_=4%, R^2^ = 20%, p < 0.05, with sex (β = 0.75, CI_95%_: 0.22–1.29, p = 0.006), SES (β = 0.45, CI_95%_: 0.06–0.85, p = 0.025), MSA (β = − 0.13, CI_95%_: −0.25 to −0.02, p = 0.025), and HIV status × push-ups (β = 0.04, CI_95%_: 0.00–0.08, p = 0.048) identified as statistically significant predictors.

### Berg Card Sorting Task

None of the 4 models used were significant predictors for BCST total correct (F’s = 0.67–1.93, p’s = 0.126–0.616). For BCST total perseverative errors, model 1 was significant, F(3, 172) = 3.15, R^2^_change_ = 5%, p < 0.05. After adding HIV status, model 2 was significant, F_change_(1, 171) = 9.78, R^2^_change_ = 6%, R^2^ = 10%, p < 0.01. Neither Model 3 nor Model 4 were significant for BCST total perseverative errors. In the final significant model, the significant predictors were HIV status (*β* = −4.26, CI_95%_: −6.94 to −1.57, p = 0.002) and SES (*β* = −3.89, CI_95%_: −6.75 to −1.04, p = 0.008).

Model 1 was significant for BCST trials to complete first set, F(3,172) = 7.86, R^2^_change_ = 12%, R^2^ = 12%, p < 0.001. Model 2, model 3, and model 4 were not significant. In Model 1, sex (β = −7.90, CI_95%_: −13.90 to −2.34, p = 0.008), age (β = 2.24, CI_95%_: 0.79–3.24, p < 0.001) and SES (β = 7.08, CI_95%_: 1.39–13.73, p = 0.025) were significant predictors for BCST Trials to complete first set. PA and Fitness information by HIV status is summarized in Table 3.

**Table 3:** PA and Fitness information by HIV status.

	Total	HIV+	HIV−
PA Aerobic (hours/week)	2.75 ± 2.32	2.44 ± 2.44	2.93 ± 2.26
PA Muscular (hours/week)	1.87 ± 2.28	1.51 ± 2.25	2.08 ± 2.28
PACER (laps)	72.43 ± 40.54	57.32 ± 37.32	80.64 ± 39.95*
push-ups (reps)	22.31 ± 9.95	19.21 ± 8.68	23.98 ± 10.21*

*p-value < 0.05 between groups independent sample t-test.

### Stroop Color Word Task

**Figure 5:**
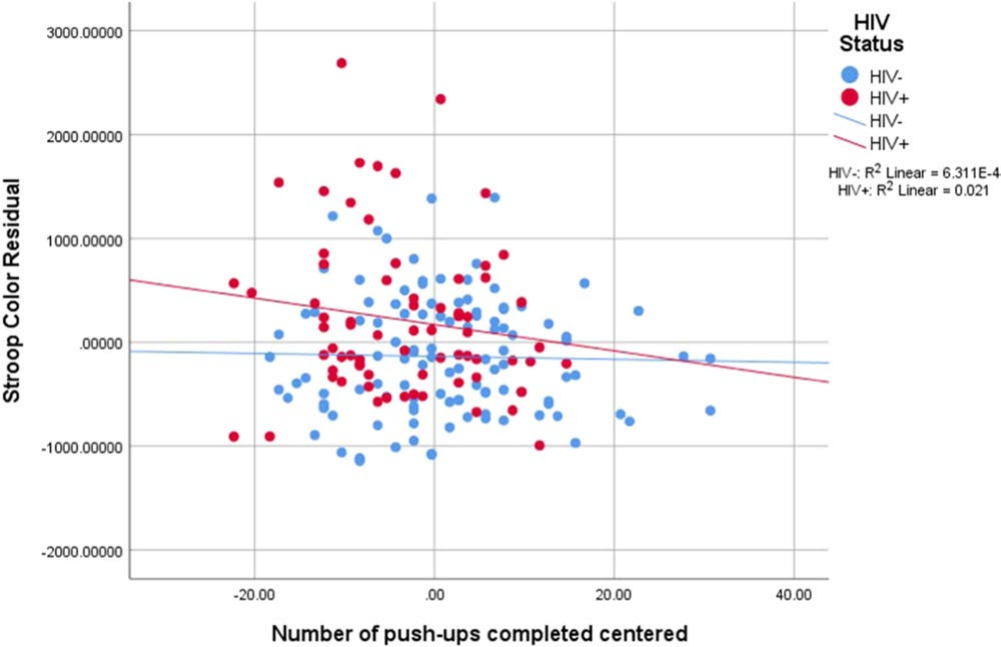
Stroop Color Total Response time residual vs. push-ups completed.

Model 1 including sex, age and SES was not a significant predictor for Stroop Color Task total response time. When including HIV status, model 2 was significant for Stroop Color Task total response time, F_change_(1, 176) = 23.69, R^2^_change_ = 12%, p < 0.001. Model 3 was not a significant predictor. With the addition of the interactions, model 4 was significant, F_change_(4, 168) = 4.08, R^2^_change_ = 7%, p < 0.01. In the final significant model, a total of 26% of the variation was explained. The significant predictors for this model were HIV status (β = 739.03, CI_95%_: 310.46–1167.60, p = 0.001) and HIV × push-ups (β = −74.70, CI_95%_: −117.61 to −31.78, p = 0.001). Figure 5 presents Stroop Color total response time residual vs. push-ups completed.

**Figure 6:**
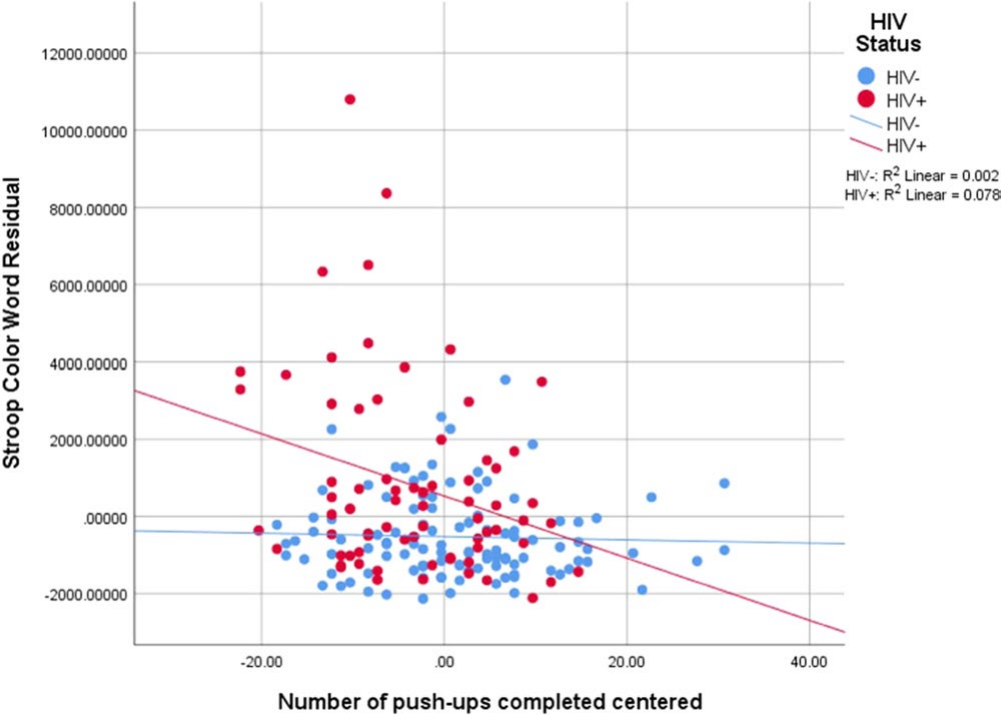
Stroop Color Word Total Response time residual vs. push-ups completed.

Model 1 was not a significant predictor for Stroop Color Word total response time. After including HIV status, model 2 was significant, F_change_(1, 176) = 25.86, R^2^_change_ = 12%, p < 0.001. Model 3 was not a significant predictor. With the inclusion of the interactions, model 4 was significant, F_change_(4, 168) = 2.88, R^2^_change_ = 5%, p < 0.05. In the final significant model, 23% of the variation in Stroop Color Word total response time was explained. Significant predictors included HIV status (β = 1134.53, CI_95%_: 526.69–1742.37, p < 0.001) and HIV × push-ups (β = −76.68, CI_95%_: −137.55 to −15.82, p = 0.014). Figure 6 presents Stroop Color Word total response time residual vs. push-ups completed.

## Discussion

This cross-sectional study is among the first to examine the relationship between HIV status, PA, fitness, and cognition in adolescents and young adults. Importantly, the results of this study consistently demonstrated that amongst children and adolescents, HIV+ status was a significant predictor of worse performance on working memory, planning, and executive functioning tasks. Given that these HIV+ individuals were on ART, these findings are disturbing because of the implications for long-term cognitive health. Fortunately, these findings also show that both HIV− and HIV+ individuals participating in MSA performed better on working memory, and that the muscular fitness of those who were HIV+ was associated with their performance on measures of working memory, information processing speed, and inhibition. In addition, sex was a significant predictor of cognitive performance such that males outperformed females on Corsi total correct, Corsi block span, and on BCST trials to complete the first set. Not surprisingly SES was a significant predictor for cognition such that a higher SES predicted better cognitive performance for both Corsi and BCST.

These results are consistent with prior findings indicating an association between exercise and cognition among HIV+ individuals and extend the current literature by showing that this relationship is present in adolescents and young adults^7,8,10,30,31^. Dufour *et al*.^7^ conducted a cross-sectional study examining the relationship between self-reported PA and performance on a neurocognitive battery of tests examining verbal fluency, working memory, speed of processing, learning and recall, executive function, and motor function. The sample consisted of young, middle-aged, and older adults (20–79 years, mean age = 47.7) and was focused on self-reported PA participation recalled over the past 72 hours. Similar to the results reported herein, the authors concluded that for HIV+ individuals, taking part in PA or exercise was related to better performance on tasks measuring working memory and speed of processing. Recently a cross-sectional study was conducted by Ortega *et al*.^8^ examining brain integrity (MRI) and cognitive functioning in physically active HIV+ middle-aged adults (mean age = 44.1 ± 17.2 and in sedentary HIV+ middle-aged adults (mean age = 40.5 ± 15.6). They found that in the physically active group, MET’s were positively correlated with executive functioning. Hence, both of these studies and the findings of this study support the potential positive relationship between PA and cognitive performance in individuals who are HIV+.

There are several limitations in the current study. The primary limitation of this study is the use of a cross-sectional design which precludes conclusions regarding cause-and-effect. However, there is substantial research evidence supporting a positive causal link between PA participation and cognitive performance by children^32,33^, young adults^34,35^, and older adults^36–39^. That being said, there is a much more limited body of evidence supporting a causal link between muscular strengthening activities and cognitive performance^40–42^. It is possible that the importance of the MSA is reflective of the particular nature of HIV which has implications for muscle wasting in advanced stages.

A second limitation of this study is that the PA measures were single-item self-reported questions that asked subjects to recall the amount of aerobic and muscular PA they participate in on a weekly basis. Although, these measures have been shown to be valid and reliable, except for MSA for Corsi block span, these measures of PA were generally found not to be predictive of cognitive performance. It is possible that these single-item questions did not provide enough sensitivity to detect relationships between PA and cognitive performance in this group of participants. However, fitness was measured objectively using the PACER and push-ups performance and results indicated that the number of push-ups that could be completed was consistently predictive of better cognitive performance for the HIV+ group. This suggests the intriguing possibility that participation in strengthening activities that result in increased upper body strength may also benefit cognitive performance. Importantly, however, because of the cross-sectional design of this study, it could also be argued that HIV+ individuals with greater cognitive performance are more likely to be fit and physically active. This may also be important because participation in PA has been shown to benefit individuals with HIV^43–45^.

This study has additional significant limitations that are worthy of consideration, despite our efforts to control for them in our analyses. First, it was not possible to match HIV+ participants and HIV− controls on age and sex. Because of logistical challenges identifying and recruiting HIV− negative participants that are an exact match to the HIV+ group on these factors, it was more practical to use age group and negative HIV test results as the basis for enrolment to the HIV− negative control group. Second, because almost all of the HIV+ participants became infected early in life (pre-natally or soon after birth) via mother-to-child transmission, premorbid cognitive functioning of the HIV+ participants could not be assessed and is largely unknown. These limitations are potential confounders of the study’s findings on age and sex differences and cognitive functioning of the HIV+ participants. Although this study does not allow for an interpretation of the direction of causality, future research could use controlled longitudinal approaches to examine if changes in fitness level or PA behaviors can improve cognitive functioning among HIV+ adolescents and young adults. The possibility of attenuating cognitive detriments associated with HAND earlier in life could represent an important area for future work.

In conclusion, the current study found that greater muscular fitness was predictive of faster speed of processing, better executive functioning, and enhanced working memory in HIV+ adolescents and young adults. As the likelihood of HAND increases with age, it is integral to find interventions that can decrease the symptoms or stave them off completely for the youth before cognitive determents become severe enough to interfere with every day living. This study adds to the current literature regarding fitness, PA, HIV status, and cognition by showing for the first time, that adolescents and young adults who engage in more PA and have greater levels of muscular fitness perform better on tasks measuring information processing, working memory, and executive functioning. The current study provides a foundation as to PA and exercise being a behavioral therapy that could possibly attenuate HAND in adolescents and young adults.
